# Infection susceptibility and immune senescence with advancing age replicated in accelerated aging *Lmna* ^*Dhe*^ mice

**DOI:** 10.1111/acel.12385

**Published:** 2015-08-07

**Authors:** Lijun Xin, Tony T. Jiang, Jeremy M. Kinder, James M. Ertelt, Sing Sing Way

**Affiliations:** ^1^ Division of Infectious DiseasesCincinnati Children's HospitalCincinnatiOH45229USA

**Keywords:** accelerated aging, infection susceptibility, models of immune senescence, progeria syndrome

## Abstract

Aging confers increased susceptibility to common pathogens including influenza A virus. Despite shared vulnerability to infection with advancing age in humans and rodents, the relatively long time required for immune senescence to take hold practically restricts the use of naturally aged mice to investigate aging‐induced immunological shifts. Here, we show accelerated aging *Lmna* ^*Dhe*^ mice with spontaneous mutation in the nuclear scaffolding protein, lamin A, replicate infection susceptibility, and substantial immune cell shifts that occur with advancing age. Naturally aged (≥20 month) and 2‐ to 3‐month‐old *Lmna* ^*Dhe*^ mice share near identically increased influenza A susceptibility compared with age‐matched *Lmna* ^*WT*^ control mice. Increased mortality and higher viral burden after influenza infection in *Lmna* ^*Dhe*^ mice parallel reduced accumulation of lung alveolar macrophage cells, systemic expansion of immune suppressive Foxp3^+^ regulatory T cells, and skewed immune dominance among viral‐specific CD8^+^ T cells similar to the immunological phenotype of naturally aged mice. Thus, aging‐induced infection susceptibility and immune senescence are replicated in accelerated aging *Lmna* ^*Dhe*^ mice.

Aging parallels an inevitable decline in protective immunity causing susceptibility to infection. This is highlighted by an estimated 40‐fold increased mortality among individuals 65 years or older after infection with ubiquitous pathogens such as influenza A virus (Thompson *et al*., 2003). This definitive indicator of human immune senescence is shared and proceeds with accelerated tempo in animals with shorter lifespans. In turn, aged mice are increasingly used to characterize immunological changes associated with advancing age. However, the expense related to maintenance and limited availability of aged mice on transgenic strain backgrounds still practically hinders mechanistic dissection of immune senescence. Herein, we propose accelerated aging phenotype mice can be exploited to bypass these limitations.

Nuclear scaffolding lamin proteins are essential for cellular homeostasis including DNA repair and chromatin structure maintenance. Reciprocally, aberrant accumulation of lamin A variants occurs in natural aging as well as accelerated aging progeria disorders (Gonzalez *et al*., 2011). In Hutchinson–Gilford syndrome, rapidly progressive loss of subcutaneous fat, joint contractures, and arteriosclerosis beginning in early childhood occurs in association with spontaneous mutations in the *LMNA* gene encoding lamin A (Hennekam, 2006; Merideth *et al*., 2008). Mice with spontaneous *Lmna* defects have similar accelerated aging phenotypes (Mounkes *et al*., 2003; Varela *et al*., 2005). For example, ‘disheveled’ *Lmna* ^*Dhe*^ mice have small stature, reduced adipose tissue, and bone mineral density caused by a point mutation in exon 1 of the murine *LMNA* homologue (Odgren *et al*., 2010). Considering the need for more practical models of immune senescence, we evaluated whether infection susceptibility and immunological shifts that occur with aging are replicated in accelerated aging *Lmna* ^*Dhe*^ mice.

In agreement with prior studies showing advancing age impairs immunity against influenza (Po *et al*., 2002; Yager *et al*., 2008), naturally aged (≥20 month) compared with young adult (2–3 month) C57Bl/6 mice were markedly more susceptible to infection. Aged mice uniformly succumbed within the first 10 days following influenza A intranasal inoculation with increased viral titers in the lung compared with young adult mice infected with either an identical or weight‐adjusted reduced inoculum (Fig. 1A,B). Susceptibility to influenza with aging was recapitulated in 2‐ to 3‐month‐old accelerated aging *Lmna* ^*Dhe*^ mice. Compared to age‐matched *Lmna* ^*WT*^ littermate controls, *Lmna* ^*Dhe*^ mice harbored significantly increased viral titers and succumbed to infection with kinetics similar to ≥20‐month‐old mice (Fig. 1A,B). Thus, aging‐induced susceptibility to influenza infection is reproduced in accelerated aging *Lmna* ^*Dhe*^ mice.

**Figure 1:**
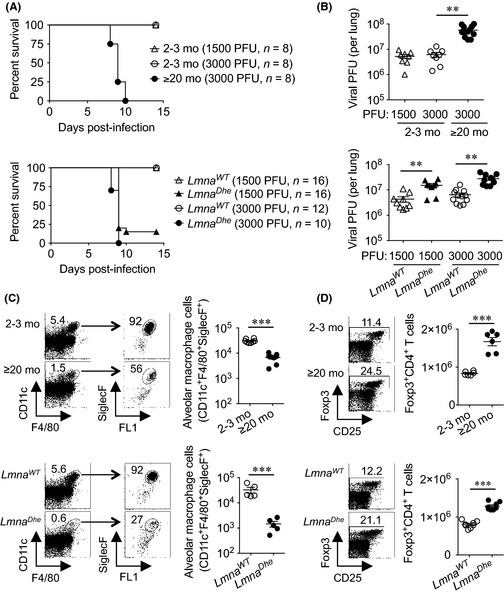
Influenza susceptibility together with skewed alveolar macrophage and regulatory T‐cell accumulation with aging are replicated in accelerated aging *Lmna* ^*Dhe*^ mice. (A) Survival of naturally aged (≥20 month) compared with 2‐ to 3‐month‐old mice (top), and 2‐ to 3‐month‐old *Lmna* ^*Dhe*^ mice compared with age‐matched *Lmna* ^*WT*^ control mice (bottom) after intranasal influenza infection. (B) Recoverable influenza viral titers day 8 postinfection for the mice described in panel A. (C) Cell gating scheme, and composite analysis of lung alveolar macrophage cells of ≥20‐month compared with 2‐ to 3‐month‐old mice (top), and 2‐ to 3‐month‐old *Lmna* ^*Dhe*^ mice compared with age‐matched *Lmna* ^*WT*^ control mice (bottom). (D) Representative FACS plots and composite analysis of Foxp3^+^ CD4^+^ splenocytes in ≥20‐month compared with 2‐ to 3‐month‐old mice (top), and 2‐ to 3‐month‐old *Lmna* ^*Dhe*^ mice compared with littermate *Lmna* ^*WT*^ control mice (bottom). These data are representative of at least two independent experiments each with similar results. Bar, mean ± 1 SE. **, *P *<* *0.01; ***, *P *<* *0.001.

Related experiments addressed whether immunological shifts implicated in causing influenza susceptibility are reiterated among naturally aged and *Lmna* ^*Dhe*^ mice. Given recent studies highlighting lung alveolar macrophage cells in innate protection early after influenza infection (Purnama *et al*., 2014; Schneider *et al*., 2014), the possibility that infection susceptibility with aging reflects reduced accumulation of these protective cells was evaluated. Sharply reduced accumulation of CD11c^+^F4/80^+^SiglecF^+^ alveolar macrophage cells was found in ≥20‐month compared with 2‐ to 3‐month‐old mice (Fig. 1C). This decline in alveolar macrophage cells with aging was reproduced, and even more prominent in *Lmna* ^*Dhe*^ compared to *Lmna* ^*WT*^ control mice (Fig. 1C). Comparatively, lung interstitial macrophage cells (CD11c^−^F4/80^+^) and dendritic cells (CD11c^+^F4/80^−^) that play non‐essential roles in protection against influenza did not differ significantly in ≥20‐month or 2‐ to 3‐month‐old *Lmna* ^*Dhe*^ mice compared with *Lmna* ^*WT*^ mice (Fig. S1). Thus, the natural decline in lung alveolar macrophage cells with aging is reproduced in accelerated aging *Lmna* ^*Dhe*^ mice, reinforcing the importance of these cells in innate antiviral immunity.

Expansion of immune suppressive regulatory T cells (Tregs) has also been causatively linked with immune senescence (Lages *et al*., 2008; Garg *et al*., 2014). In agreement, CD4^+^ splenocytes that co‐express the Treg defining marker, Foxp3, were expanded in ≥20‐month compared with 2–3‐month‐old mice (Fig. 1D). Aging‐induced accumulation of Tregs was recapitulated in *Lmna* ^*Dhe*^ mice that contain significantly increased frequency and number of Foxp3^+^ CD4^+^ T cells compared with *Lmna* ^*WT*^ controls (Fig. 1D). Foxp3^+^ cells in aged compared with *Lmna* ^*Dhe*^ mice also showed similar shifts in expression of some (e.g., CD103, ICOS), but not all Treg cell‐intrinsic functional markers (Vignali *et al*., 2008) (Fig. S2). Further analysis of Foxp3^+^ CD4^+^ T cells derived from *Lmna* ^*Dhe*^ donor bone marrow progenitors shows reconstitution in *Lmna* ^*WT*^ recipient mice efficiently eliminates quantitative expansion and qualitative shifts for *Lmna* ^*Dhe*^ Tregs (Fig. S3). Together with T‐cell‐extrinsic factors driving severe lymphopenia when lamin A is completely eliminated in *Lmna*‐deficient mice (Hale *et al*., 2010), these results indicate nonessential cell‐intrinsic roles for lamin A in peripheral CD4^+^ T‐cell homeostasis.

To investigate changes in other adaptive immune components associated with advancing age, CD8^+^ T‐cell dynamics prior to and expansion after influenza infection were evaluated in *Lmna* ^*Dhe*^ mice. Here, declining numbers of peripheral CD8^+^ T cells, their ratio to CD4^+^ T cells, and qualitative shifts favoring memory and exhaustion phenotypes (e.g., CD44^hi^, PD‐1^hi^) previously reported with aging were reproduced in our analysis of ≥20‐month compared with 2‐ to 3‐month‐old mice (Fig. S4) (Decman *et al*., 2012). Interestingly, however, with the exception of modestly increased CD44 and PD‐1 expression, these CD8^+^ T‐cell‐intrinsic shifts were not observed in *Lmna* ^*Dhe*^ compared with littermate *Lmna* ^*WT*^ control mice (Fig. S4).

Extending this analysis to mediastinal lymph node cells after influenza infection did not show significant differences in IFN‐γ production by CD8^+^ T cells of ≥20‐month compared to 2‐ to 3‐month‐old mice in response to NP_336–374_ peptide stimulation (Fig. 2A). Comparatively, IFN‐γ production by CD8^+^ T cells from aged mice and the ratio of IFN‐γ‐producing CD8^+^ T cells between aged compared with young adult mice were significantly increased after PA_224–233_ peptide stimulation (Fig. 2A and S5). Importantly, regardless of stimulation with NP_336–374_ or PA_224–233_ peptide under our experimental conditions, the accumulation and ratio of IFN‐γ‐producing influenza‐specific CD8^+^ T cells from *Lmna* ^*Dhe*^ compared with *Lmna* ^*WT*^ controls directly parallel results for aged compared to young adult mice (Figs 2A and S5).

**Figure 2:**
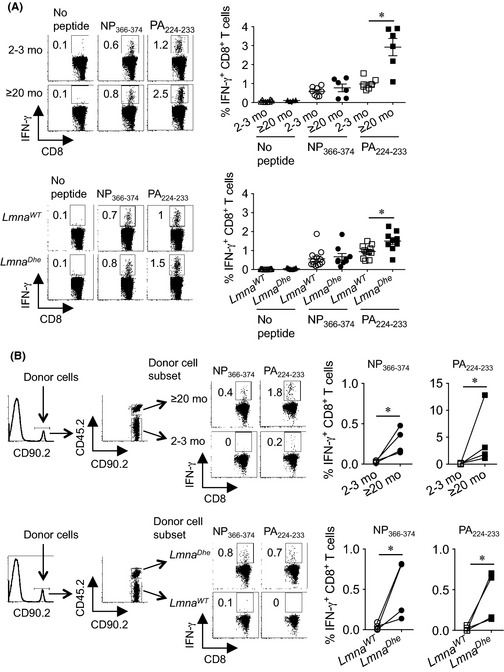
Skewed immune dominance of influenza virus‐specific CD8^+^ T cells with aging is replicated in accelerated aging *Lmna* ^*Dhe*^ mice. (A) Representative FACS plots and composite analysis of percent IFN‐γ‐producing mediastinal lymph node CD8^+^ T cell after stimulation with each influenza‐specific MHC class I peptide compared with no stimulation controls day 8 after influenza A infection (3000 PFUs) in ≥20‐month compared with 2‐ to 3‐month‐old mice (top), or 2‐ to 3‐month‐old *Lmna* ^*Dhe*^ mice compared with littermate *Lmna* ^*WT*^ control mice (bottom). (B) Cell gating scheme, and composite analysis of percent IFN‐γ‐producing CD8^+^ splenocytes after stimulation with PA_224–233_ or NP_366–374_ peptides for ≥20‐month compared with 2‐ to 3‐month‐old donor mice (top), or 2‐ to 3‐month‐old *Lmna* ^*Dhe*^ mice compared with *Lmna* ^*WT*^ donor mice (bottom) day 8 after influenza A infection (3000 PFUs). These data are representative of results from at least two independent experiments with similar results. Bar, mean ± 1 SE. *, *P *<* *0.05.

To dissociate whether more robust PA_224–233_‐specific CD8^+^ T‐cell expansion in aged and *Lmna* ^*Dhe*^ mice reflects increased viral burden (Figs 1B) or cell‐intrinsic changes associated with aging (Yager *et al*., 2008; Decman *et al*., 2012), accumulation of IFN‐γ‐producing CD8^+^ T cells from each group of donor mice was compared after adoptive transfer into congenically discordant naive recipients (Fig. 2B). Remarkably, this analysis showed consistently increased expansion of PA_224–233_ as well as NP_336–374_‐specific CD8^+^ T cells from ≥20‐month and *Lmna* ^*Dhe*^ mice, each in comparison with cotransferred donor CD8^+^ T cells from 2‐ to 3‐month‐old *Lmna* ^*WT*^ mice (Fig. 2B). Thus, cell‐extrinsic factors shared by naturally aged and *Lmna* ^*Dhe*^ mice selectively restrict expansion of influenza NP_366–374_‐specific CD8^+^ T cells promoting skewed immune dominance with aging that favors expansion of PA_224–233_ to NP_366–374_ influenza‐specific CD8^+^ T cells (Po *et al*., 2002; Yager *et al*., 2008; Jiang *et al*., 2009).

Despite an increasing assortment of immunological shifts recognized to occur with aging (Dorshkind & Swain, 2009), the specific changes responsible for functional immune senescence with ensuing infection susceptibility remain largely undefined. A prohibitive roadblock using mouse infection models stems from long‐time intervals required for declining immunity to take hold (≥15–20 months) (Lages *et al*., 2008; Decman *et al*., 2012). To circumvent this limitation, we reasoned accelerated aging mouse models can be used to more efficiently unravel the key aging‐induced immunological shifts that control infection susceptibility. Although a variety of mice that display premature aging caused by spontaneous or engineered genetic defects have been described (Liao & Kennedy, 2014), this is the first reported use of any accelerated aging strain to probe aging‐induced infection susceptibility. We show influenza susceptibility that occurs with natural aging is faithfully reproduced in progeroid *Lmna* ^*Dhe*^ mice. Shifts in many innate and adaptive immune components implicated in causing influenza A susceptibility or diminished immunity with advancing age are also faithfully reproduced in *Lmna* ^*Dhe*^ mice. In particular, conserved decline in alveolar macrophage cells and expansion of immune suppressive Foxp3^+^ Tregs that parallel influenza susceptibility in naturally aged and *Lmna* ^*Dhe*^ mice suggest these immune cell shifts play dominant roles impairing host defense with advancing age. On the other hand, the discordance in CD8^+^ T‐cell numerical decline, along with expression of memory and exhaustion markers by these cells, between aged and *Lmna* ^*Dhe*^ mice suggests shifts in these immune components play less pivotal roles promoting influenza susceptibility. Additional studies are required to investigate whether these findings extend to aging‐induced susceptibility to pathogens that cause more persistent infection (Lages *et al*., 2008), and the use of accelerated aging mice to dissect the immune pathogenesis of aging increasingly recognized to parallel latent infection with other viral pathogens (Lages *et al*., 2008; Nikolich‐Zugich, 2014). Nonetheless, the fidelity whereby progeroid *Lmna* ^*Dhe*^ mice reproduce influenza susceptibility that occurs with advancing age highlights accelerated aging mice as important complementary tools for further dissecting the fundamental biology of aging‐induced immune senescence.

## Materials and methods

### Mice

C57Bl/6 (B6) mice (2–3 months old), congenic B6.SJL‐*Ptprc* ^*a*^ (CD45.1^+^), and B6.PL‐*Thy1* ^*a*^ (CD90.1^+^) were purchased from The Jackson Laboratory (Bar Harbor, ME, USA). Naturally aged (≥20 month old) B6 mice were provided by The National Institute on Aging (Bethesda, MD, USA). *Lmna* ^*Dhe*^ and *Lmna* ^*WT*^ littermate control mice on B6 background were purchased from The Jackson Laboratory, bred in our specific pathogen free animal facility, and analyzed at 2–3 month of age. For generating mixed bone marrow chimeric mice, bone marrow progenitors cells (1 × 10^6^) from congenically discordant *Lmna* ^*Dhe*^ (CD45.2^+^CD90.2^+^) and *Lmna* ^*WT*^ (CD45.2^+^CD90.1^+^) mice were combined at a 1:1 ratio, intravenously transferred into sublethally irradiated twice‐daily (550 rads) CD45.1^+^ recipients, and analyzed after 8–10 weeks of reconstitution. For adoptive transfer, mononuclear cells from the spleen plus peripheral lymph nodes of ≥20‐month‐aged (CD90.2^+^CD45.2^+^) and 2‐ to 3‐month‐old (CD90.2^+^CD45.2^−^) mice were combined at a 1:1 ratio (10^8^ cells from each donor) and intravenously transferred into CD90.2^−^CD90.1^+^ naive young adult recipient mice one day prior to influenza infection. All experiments were performed under Cincinnati Children's Hospital Institutional Animal Care and Use Committee approved protocols.

### Influenza infection

The PR8 strain of influenza A virus (A/Puerto Rico/34, H1N1) was provided by Dr. Monica M. McNeal (Cincinnati Children's Hospital). For infection, mice were anaesthetized intraperitoneally with ketamine (100 mg kg^−1^) and xylazine (10 mg kg^−1^) and intranasally inoculated with 1500 or 3000 plaque‐forming units (PFUs) of PR8 virus suspended in 30 μL endotoxin‐free saline. For enumerating recoverable influenza PFUs after infection, the lungs of each group of mice were sterilely dissected, homogenized in saline, and tittered in triplicate onto Madin–Darby canine kidney cell monolayers. After incubation with the lung homogenate for 18 hours, viral plaques were detected using a monoclonal mouse anti‐influenza A antibody (M4090913; Fitzgerald, Acton, MA, USA) followed by staining with FTIC‐conjugated goat anti‐mouse IgG antibody (AQ303F; Millipore, Billerica, MA, USA).

### Flow cytometry

The following antibodies for cell surface, intracellular, or intranuclear staining were purchased from eBioscience (San Diego, CA, USA) or BD Biosciences (San Jose, CA, USA): anti‐CD4 (GK1.5), anti‐CD8a (53–6.7), anti‐CD25 (PC61), anti‐CD45 (30‐F11), anti‐CD11c (N418), anti‐SiglecF (E50‐2440), anti‐F4/80 (BM8), anti‐CTLA‐4 (UC10‐4B9), anti‐GITR (DTA‐1), anti‐ICOS (7E.17G9), anti‐OX40 (OX86), anti‐CD103 (2E7), anti‐Helios (22F6), anti‐Foxp3 (FJK‐16s), anti‐CD44 (IM7), anti‐CD62L (MEL‐14), anti‐CD69 (H1.2‐F3), anti‐CD127 (A7R34), anti‐KLRG1 (2F1), anti‐LAG3 (ebioC9B7W), anti‐PD‐1 (J43), CD90.2 (30‐H12), anti‐CD45.2 (104), anti‐IFN‐γ (XMG1.2). For intranuclear Foxp3 staining, surface stained cells were fixed and permeabilized using commercially available reagents (eBioscience) and stained with anti‐Foxp3 antibody. For cytokine production, single‐cell suspensions of mediastinal lymph node cells were stimulated with 10 μm of influenza A‐specific peptides NP_366–374_ (ASNENMETM) or PA_224–233_ (SSLENFRAYV) for 6 hours in the presence of GlogiPlug™ (BD Biosciences) compared with no stimulation controls and subjected to intracellular cytokine staining using commercially available reagents (BD Biosciences) and anti‐IFN‐γ. Flow cytometry samples were acquired on a BD FACSCanto^™^ cytometer and analyzed using FlowJo software (Tree Star, Ashland, OR, USA).

### Isolation of lung tissue cells

The lungs were first cut into small pieces (0.1–0.2 cm) and then incubated in medium supplemented with 10% FBS, 500 μg mL^−1^ collagenase D, 50 μg mL^−1^ DNase I, and 500 μg mL^−1^ dispase for 60 min with gentle agitation. Thereafter, the lung homogenate was passed through a mesh filter (100 μm) and centrifuged on Percoll gradient (40% and 70%) for 20 min (530 *g*). Thereafter, each subset of cells (CD11c^+^F4/40^+^SiglecF^+^ alveolar macrophage cells, CD11c^−^F4/40^+^ interstitial macrophage cells, and CD11c^+^F4/40^−^ dendritic cells) were identified among total mononuclear CD45^+^ leukocyte cells.

### Statistics

The differences in survival after infection between groups of mice were analyzed using the log‐rank (Mantel–Cox) test. The differences in recoverable viral PFUs and cell number between groups of mice were analyzed using the Student's *t*‐test (two groups) or anova with Dunnett correction for multiple comparisons (more than two groups), with *P *<* *0.05 taken as statistical significance.

## Conflict of interest

The authors have no conflicts of interest to declare.

## Supporting information


**Fig. S1** Similar numbers of interstitial macrophage and dendritic cells in the lungs of naturally aged and accelerated aging *Lmna* ^*Dhe*^ mice.

**Fig. S2** Expression of regulatory T cell‐intrinsic phenotypic markers in naturally aged and *Lmna* ^*Dhe*^ mice.

**Fig. S3** Cell‐extrinsic factors drive Foxp3^+^ T cell shifts in *Lmna* ^*Dhe*^ mice.

**Fig. S4** Discordant accumulation and phenotypical shifts for CD8^+^ T cells between naturally aged and *Lmna* ^*Dhe*^ mice.

**Fig. S5** Selectively increased expansion of influenza PA_224–233_ compared with NP_366–374_‐specific CD8^+^ T cell from naturally aged and accelerated aging *Lmna* ^*Dhe*^ mice.
