# Supplementary material for: Infection susceptibility and immune senescence with advancing age replicated in accelerated aging Lmna Dhe mice
Source: Aging Cell. 2015 Aug 7;14(6):1122–6. doi: 10.1111/acel.12385 (PMC4693468; doi:10.1111/acel.12385)
Supplement: Supplementary file 1 — Fig. S1 Similar numbers of interstitial macrophage and dendritic cells in the lungs of naturally aged and accelerated aging Lmna Dhe mice. [file ACEL-14-1122-s001.pdf]

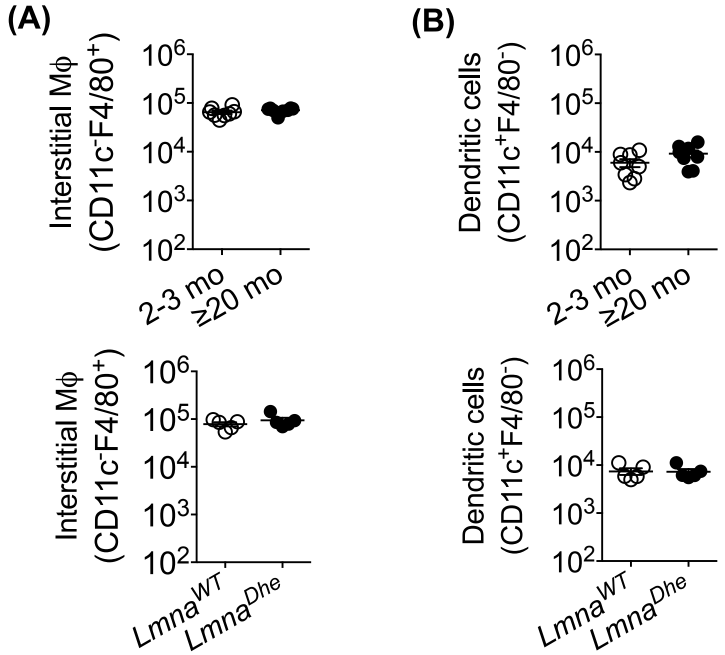

Supplementary Figure 1. Similar numbers of interstitial macrophage and dendritic cells in the lungs of naturally aged and accelerated aging *Lmna*^{Dhe} mice. (**A**) Number of interstitial macrophage (F4/80^{+}CD11c^{-}) cells in the lungs of ≥ 20 month compared with 2-3 month old mice (top), and 2-3 month old *Lmna*^{Dhe} mice compared with age-matched *Lmna*^{WT} mice (bottom). (**B**) Number of dendritic cells (DC11c^{+}F4/80^{-}) cells in the lungs of ≥ 20 month compared with 2-3 month old mice (top), and 2-3 month old *Lmna*^{Dhe} mice compared with age-matched *Lmna*^{WT} mice (bottom). These data are representative of two independent experiments each containing 3-4 mice per group. Bar, mean ± one SE.
