# Supplementary material for: Infection susceptibility and immune senescence with advancing age replicated in accelerated aging Lmna Dhe mice
Source: Aging Cell. 2015 Aug 7;14(6):1122–6. doi: 10.1111/acel.12385 (PMC4693468; doi:10.1111/acel.12385)
Supplement: Supplementary file 3 — Fig. S3 Cell‐extrinsic factors drive Foxp3+ T cell shifts in Lmna Dhe mice. [file ACEL-14-1122-s003.pdf]

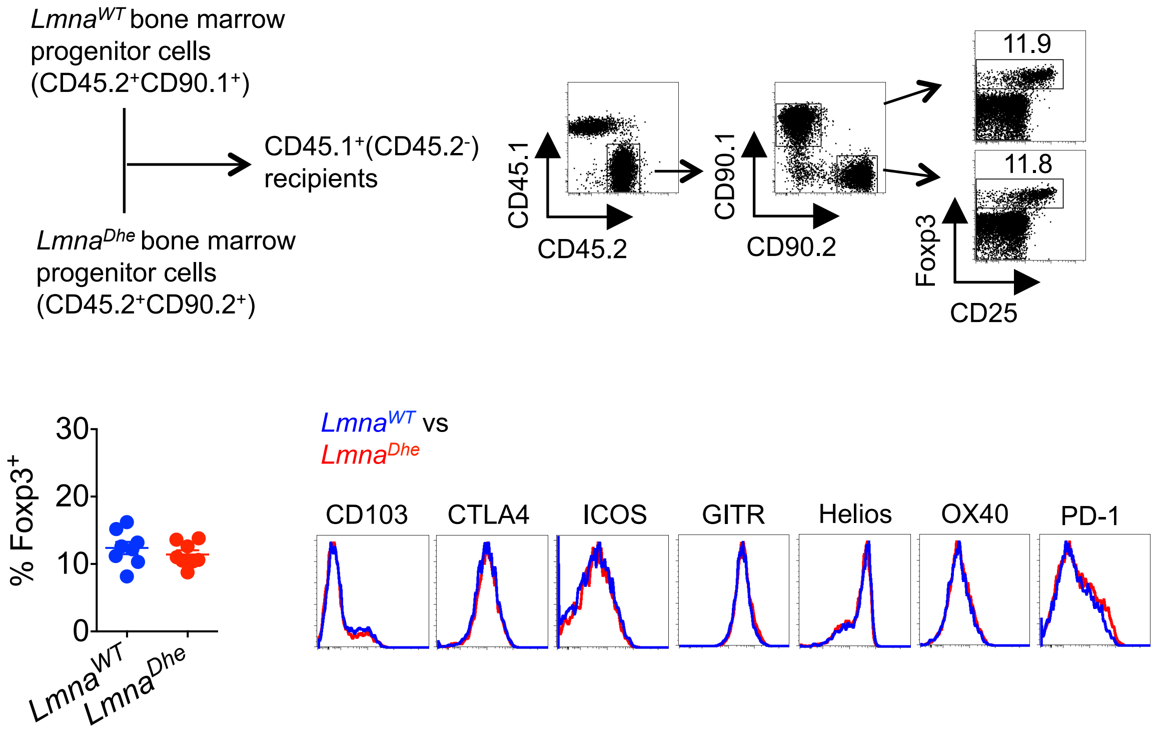

Supplementary Figure 3. Cell-extrinsic factors drive Foxp3^{+} T cell shifts in *Lmna^{Dhe}* mice. Representative plots show the gating scheme for CD4^{+} cells (top), along with composite analysis of percent Foxp3^{+} among CD4^{+} splenocytes (bottom left) and expression of each marker by *Lmna^{WT}* (blue histograms) compared with *Lmna^{Dhe}* (red histograms) Foxp3^{+} CD4^{+} T cells (bottom right) in mixed chimera mice 8-10 weeks after reconstitution. These data are representative of at least two independent experiments each containing 4 mice per group with similar results. Bar, mean ± one SE.
