# Supplementary material for: Infection susceptibility and immune senescence with advancing age replicated in accelerated aging Lmna Dhe mice
Source: Aging Cell. 2015 Aug 7;14(6):1122–6. doi: 10.1111/acel.12385 (PMC4693468; doi:10.1111/acel.12385)
Supplement: Supplementary file 4 — Fig. S4 Discordant accumulation and phenotypical shifts for CD8+ T cells between naturally aged and Lmna Dhe mice. [file ACEL-14-1122-s004.pdf]

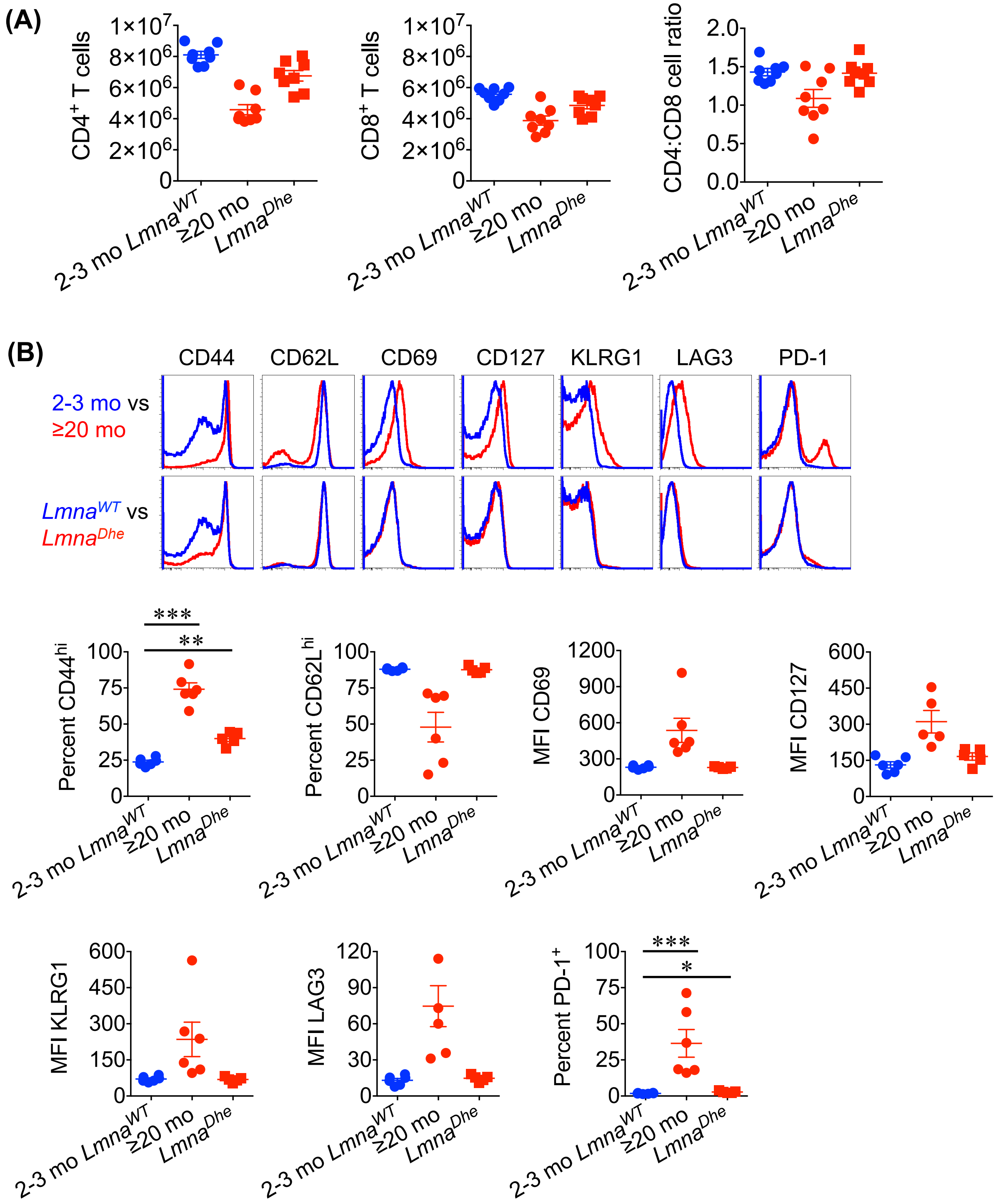

Supplementary Figure 4. Discordant accumulation and phenotypical shifts for $CD8^+$ T cells between naturally aged and *Lmna*^{Dhe} mice. (**A**) Number of $CD4^+$ and $CD8^+$ splenocytes, and ratio of $CD4^+$ to $CD8^+$ cells from ≥ 20 month old and 2-3 month old *Lmna*^{Dhe} mice compared with 2-3 month old *Lmna*^{WT} mice. (**B**) Representative histogram plots and composite analysis of percent positive or mean fluorescence intensity (MFI) for expression of each marker by $CD8^+$ T cells for the mice described in panel A. These data are representative of results from at least two independent experiments each containing 3-4 mice per group with similar results. Bar, mean ± one SE. *, $P < 0.05$; **, $P < 0.01$.
