# Supplementary material for: Infection susceptibility and immune senescence with advancing age replicated in accelerated aging Lmna Dhe mice
Source: Aging Cell. 2015 Aug 7;14(6):1122–6. doi: 10.1111/acel.12385 (PMC4693468; doi:10.1111/acel.12385)
Supplement: Supplementary file 5 — Fig. S5 Selectively increased expansion of influenza PA224–233 compared with NP366–374‐specific CD8+ T cell from naturally aged and accelerated aging Lmna Dhe mice. [file ACEL-14-1122-s005.pdf]

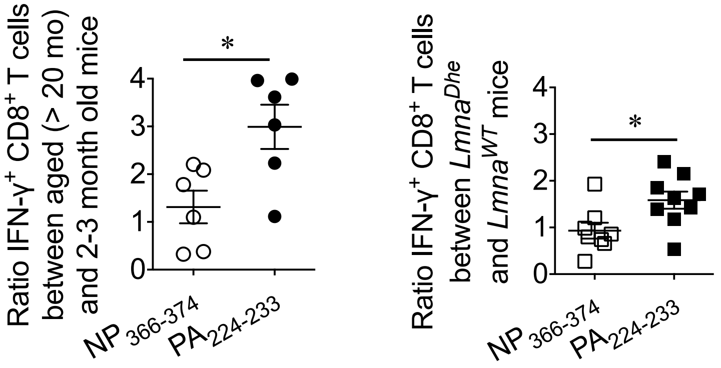

Supplementary Figure 5. Selectively increased expansion of influenza $PA_{224\text{-}233}$ compared with $NP_{366\text{-}374}$-specific $CD8^+$ T cell from naturally aged and accelerated aging *Lmna*^{Dhe} mice. Ratio of IFN-γ producing $CD8^+$ T cells after stimulation of with each influenza MHC class I peptide day 8 after influenza A infection (3000 PFUs) in ≥ 20 month compared with 2-3 month old mice (left), and 2-3 month old *Lmna*^{Dhe} mice compared with age-matched *Lmna*^{WT} mice (right). These data are representative of results from at least two independent experiments with similar results. Bar, mean ± one SE.
