# Supplementary material for: Infection susceptibility and immune senescence with advancing age replicated in accelerated aging Lmna Dhe mice
Source: Aging Cell. 2015 Aug 7;14(6):1122–6. doi: 10.1111/acel.12385 (PMC4693468; doi:10.1111/acel.12385)
Supplement: Supplementary file 2 — Fig. S2 Expression of regulatory T cell‐intrinsic phenotypic markers in naturally aged and Lmna Dhe mice. [file ACEL-14-1122-s002.pdf]

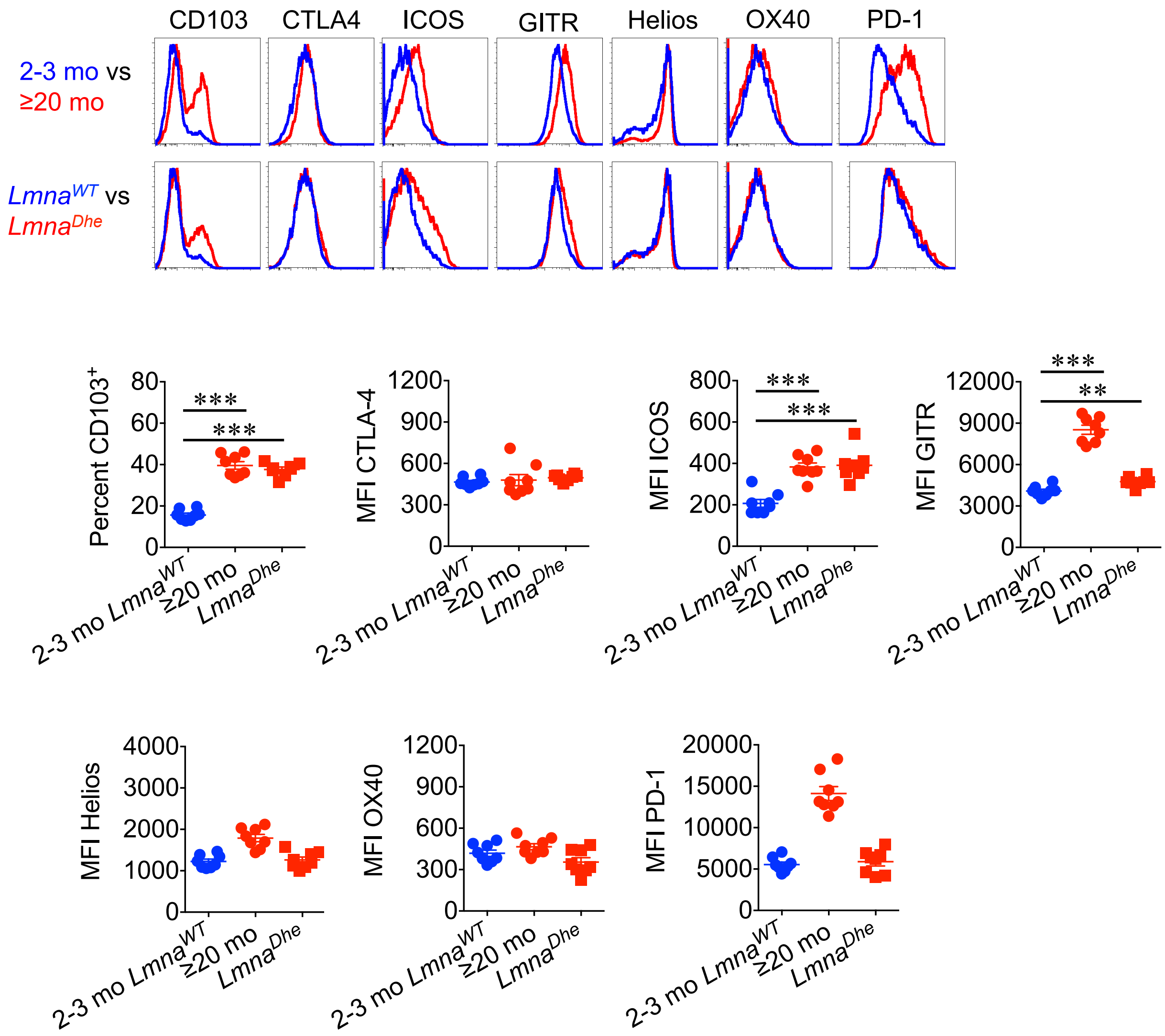

Supplementary Figure 2. Expression of regulatory T cell-intrinsic phenotypic markers in naturally aged and *Lmna*^{Dhe} mice. Representative histogram plots (top) and composite analysis of percent positive or mean fluorescence intensity (MFI) (bottom) for expression of each marker by Foxp3^{+} CD4^{+} splenocytes of ≥ 20 month (top) and 2-3 month old *Lmna*^{Dhe} mice (bottom) compared with 2-3 month old *Lmna*^{WT} mice. These data are representative of at least two independent experiments each containing 4 mice per group with similar results. Bar, mean ± one SE.
